# Selective Enhancement of Surface and Bulk E-Field within Porous AuRh and AuRu Nanorods

**DOI:** 10.1021/acs.jpcc.1c08699

**Published:** 2021-12-12

**Authors:** Joshua Piaskowski, Alisher Ibragimov, Fedja J. Wendisch, Gilles R. Bourret

**Affiliations:** Department of Chemistry and Physics of Materials, University of Salzburg, Jakob Haringer Strasse 2A, A-5020 Salzburg, Austria

## Abstract

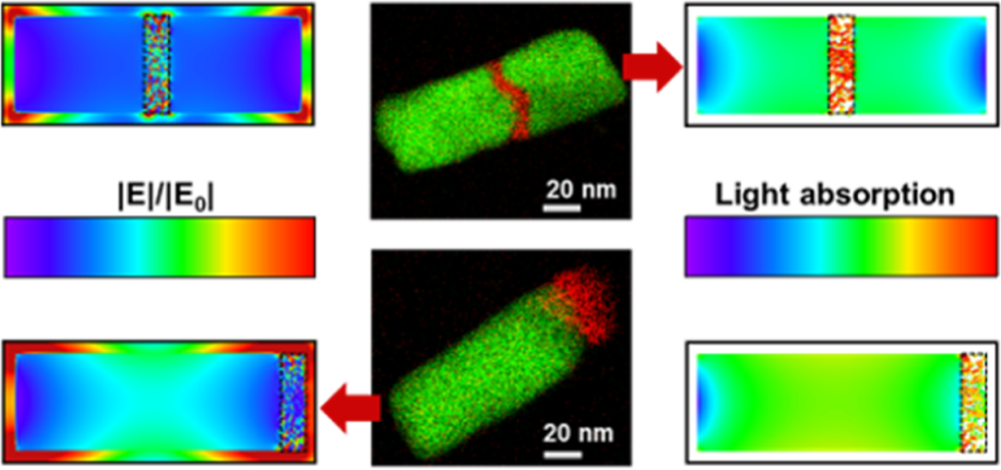

A variety of multisegmented nanorods (NRs) composed of dense Au and porous Rh and Ru segments with lengths controlled down to ca. 10 nm are synthesized within porous anodic aluminum oxide membranes. Despite the high Rh and Ru porosity (i.e., ∼40%), the porous metal segments are able to efficiently couple with the longitudinal localized surface plasmon resonance (LSPR) of Au NRs. Finite-difference time-domain simulations show that the LSPR wavelength can be precisely tuned by adjusting the Rh and Ru porosity. Additionally, light absorption inside Rh and Ru segments and the surface electric field (E-field) at Rh and Ru can be independently and selectively enhanced by varying the position of the Rh and Ru segment within the Au NR. The ability to selectively control and decouple the generation of high-energy, surface hot electrons and low-energy, bulk hot electrons within photocatalytic metals such as Rh and Ru makes these bimetallic structures great platforms for fundamental studies in plasmonics and hot-electron science.

## Introduction

The excitation of localized surface plasmon resonances (LSPRs) within metal nanoparticles can lead to very large enhancements of the electric field (E-field) inside and on the nanoparticle.^1−4^ Such E-field enhancements have been used for many applications, such as surface-enhanced Raman scattering,^1,5,6^ hot-electron-induced photodetection,^2^ plasmonic heating,^7^ biosensing,^8^ plasmon-modulated light emission,^9,10^ and more recently plasmon-enhanced photocatalysis.^11−19^

Metal nanoparticles can increase reaction rates and selectivity under light irradiation via the generation of energetic “hot” charge carriers that can be directly or indirectly transferred into adsorbates or thermally relax and provide a localized increase in temperature.^3,7,11,13,19−22^ These fascinating processes make them great candidates to engineer further important and complex catalytic reactions. Theoretical works have suggested that “high-energy” electrons are generated at the metal surface with a rate proportional to the normal component of |*E*|^2^ at the metal surface, while lower-energy electrons are generated inside the NP with a rate that depends on bulk absorption and thus on |*E*|^2^ inside the NP (i.e., integrated over the NP volume).^2,14,23^ To date, it is still unclear which type of hot electrons plays a dominant role, while the effect of plasmonic heating is still under study.^24−28^ Thus, the design and study of platforms providing an independent control over the bulk and surface E-field at lossy but efficient photocatalysts would be welcome by the community.

**Figure 1:**
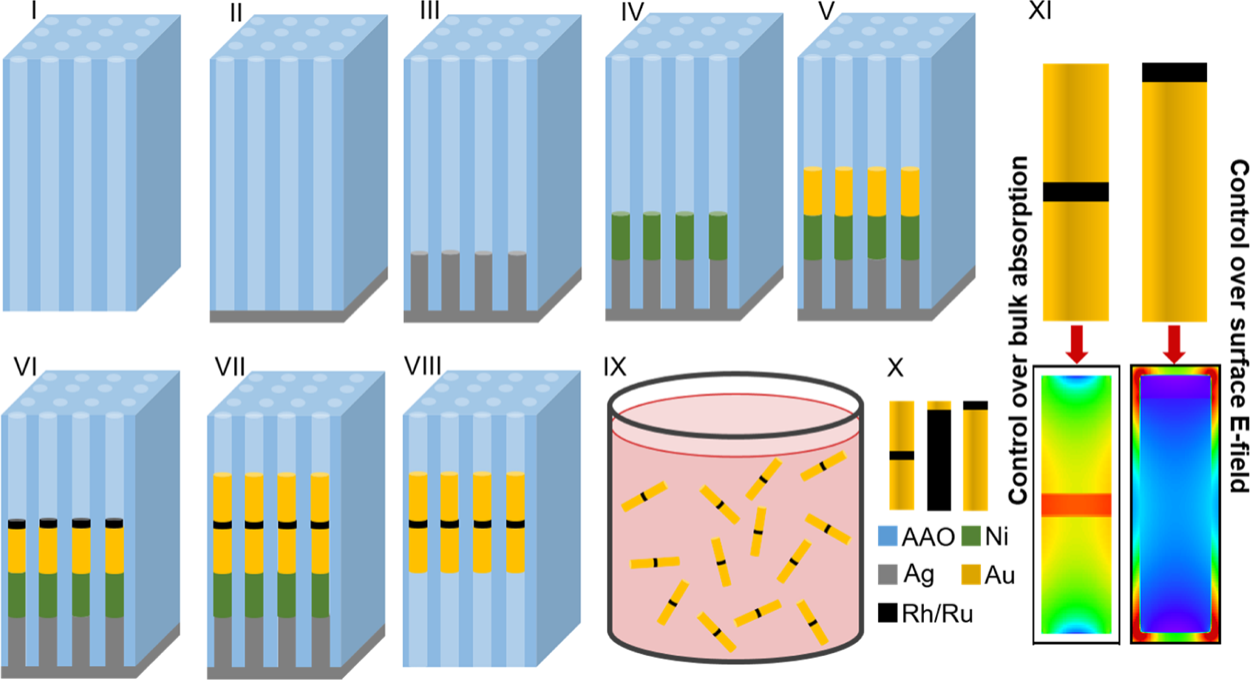
Schematic synthesis of heterometallic NRs within tubular pores of anodic aluminum oxide membranes. (I) Cross section of an AAO membrane. (II) One side of the membrane is coated with Ag by sputter deposition. (III) Electrodeposition of a sacrificial Ag segment. (IV) Electrodeposition of a protective Ni segment to avoid galvanic exchange reactions between Au and Ag. (V–VII) Subsequent electrodeposition of the desired structure (here: AuRhAu or AuRuAu). (VIII) Etching of the Ag film and Ag and Ni segments using HNO_3_. (IX) Release of the heterometallic NRs by dissolution of the aluminum oxide membrane in NaOH. (X) Structures synthesized during this work: AuRhAu and AuRuAu; AuRh with long Rh segments; and AuRh and AuRu with long Au segments. (XI) Selective enhancement of either light absorption in the bulk or surface E-field by controlling the Rh and Ru segment location within the bimetallic NRs.

Herein, we report the synthesis of bimetallic nanorods (NRs) composed of a plasmonic gold absorber and a porous lossy metal segment, composed of either Rh or Ru, via templated electrodeposition within porous anodic aluminium oxide (AAO) membranes (Figure 1). The approach, pioneered by Martin, Penner, and Moskovits,^29−31^ provides a powerful way to synthesize a large variety of multisegmented metal and semiconductor nanowires with a high control over the segment length, composition, and morphology.^5,9,32−36^ Because the segment length is electrochemically controlled, templated deposition within AAO membranes can provide a spatial resolution down to the sub-5 nanometer range^5,35^ that is well-suited for the combination of different materials at the nanoscale.

Rh and Ru were selected as the lossy metals because of their high relevance for light-enhanced selectivity and reaction rate, previously demonstrated for CO_2_ photomethanation.^37,38^ Unlike Au that is a canonical material for plasmonics with strong LSPRs, Rh and Ru suffer from large losses in the visible and near-IR range. This is because ε″, the imaginary part of the metal dielectric function responsible for light absorption, is relatively low for Au, while it is much higher for Rh and Ru (Figure S1).^39−41^ The losses associated with a high ε″ significantly dampen the LSPR, effectively lowering the field enhancement in the nanoparticle at the LSPR. Additionally, while porous plasmonic nanostructures composed of Au and Ag provide enhanced E-fields compared to their nonporous counterpart,^42−44^ porous lossy metals yield low enhancements.

To mitigate this, thin segments of porous Rh and Ru were integrated within Au NRs. A dramatic increase in surface and bulk E-field enhancement with the porous metal is demonstrated by our electromagnetic simulations based on the finite-difference time-domain (FDTD) method. The high synthetic control afforded by our templated electrodeposition method was used to investigate the effect of Rh and Ru segment location and porosity on their optical coupling with the Au NR longitudinal LSPR. A spatioselective control over bulk and surface E-field enhancement of the lossy metal segment is supported by our FDTD simulations, both of which regulate the energy and generation rate of hot electrons.^2,14,17,23,45,46^

## Experimental Methods

### NR Fabrication

Heterometallic segmented NRs were electrochemically deposited in the pores of anodized aluminum oxide (AAO) membranes (35 and 55 nm nominal pore diameter, 25 mm membrane diameter, and 50 μm thickness, Synkera Technologies Inc.) using a three-electrode setup (platinum counter electrode; Ag/AgCl reference electrode). Before electrochemical deposition, one side of the AAO membrane was sputter-coated with a 100 nm-thick Ag layer. As the first segment, a long Ag segment was plated into the pores from a Technic Silver Cy Less II W RTU solution at a constant potential of −0.94 V versus Ag/AgCl. Subsequently, Ni was grown from a Technic Nickel Sulfamate RTU solution at a constant potential of −1.1 V versus Ag/AgCl. After this, the desired structure could be grown on top. Ru was grown from a ruthenium U plating solution from Technic at a constant potential of −0.65 V versus Ag/AgCl, and Au was deposited from a Technic Orotemp 24 S solution at a constant potential of −0.95 V versus Ag/AgCl. Rh was deposited from a homemade aqueous solution made of 10 mM RhCl_3_ and 0.5 M NaCl at a constant potential of −0.35 V versus Ag/AgCl.

After the heterometallic NR structure was built within the pores, the Ag layer and NRs were etched by immersing the membrane in a mixture of EtOH (96%), NH_4_OH (25%), and H_2_O_2_ (30%) and with a ratio of 4:1:1 for 20 h. After 2 h, the solution was renewed for the residual 18 h. Subsequently, the Ni segments were etched using 10 wt % HNO_3_. Afterward, the desired heterometallic NR structure is released by dissolving the AAO membrane in an aqueous 3 M NaOH solution with 0.1 wt % trisodiumcitrate. Subsequently, the NRs are rinsed three times by centrifuging with 6000 rpm acceleration and solvent exchange to 0.1 wt % aqueous trisodiumcitrate solution.

UV–vis spectra were acquired using a PerkinElmer Lambda 750 UV/Vis spectrometer with a 3D WB detector. The spectra were corrected so that a continuous curve over the change in the detector and source (at 860 nm) was obtained. Without this correction, a drop of ±10% in the extinction spectra was visible at this wavelength.

Scanning transmission electron microscopy (STEM) analysis was carried out using a cold field emission gun JEOL F200 STEM/TEM operated at 200 kV accelerating voltage, with a probe diameter of 0.16 nm and a probe current of 0.1 nA. EDX maps were obtained using a large windowless JEOL Centurio EDX detector (100 mm^2^, 0.97 sr, and energy resolution < 133 eV) by integrating the counts over a specific transition:

Au M_α_ integrated between 2.02 and 2.22 keV.

Rh L_α_ integrated between 2.44 and 2.68 keV.

Ru L_α_ integrated between 2.58 and 2.81 keV.

### FDTD Simulations

FDTD simulations were carried out using the commercial FDTD package from Lumerical Inc. Single NRs were irradiated with a total field-scattered field source in the 200–2000 nm wavelength range. The incident light was linearly polarized. The NRs were aligned along the *z*-axis. Simulations were performed for three different incident wave vectors *k*: two wave vectors being injected along the *y*-axis with E-field polarization along the *x*- and *z*-axis and one wave vector injected along the *z*-axis, polarized along the *x*-axis. Extinction spectra were calculated by averaging over the sum of scattering and absorption spectra of the different polarizations/NR orientations. The required material properties from the metals were used from the Lumerical materials library. Palik’s data were used for rhodium^39^ and Johnson and Christy’s data for gold.^41^ The optical data for ruthenium was reproduced from Palik.^40^ The refractive index of the surrounding medium was set to a constant value of 1.33. Typical resolutions of the simulations was 0.35 nm mesh size around porous structures and 1.0 nm mesh size around the dense metal segments. The absorbed power in a mesh cell inside the metal was calculated as
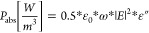
where ε_0_ is the dielectric constant of the surrounding media, ω is the angular frequency, and *E* is the E-field intensity.

To mimic the porosity and nanocrystalline structure of Rh and Ru segments, spheres with a 2 nm diameter were randomly positioned in the cylindrical volume of the NR segment. This was done by generating random numbers for the *x*, *y*, *z* coordinates of the spheres. To avoid the formation of a dense structure, the maximum overlap of the spheres was controlled, by only adding a sphere into the segment if the center-to-center distance to each neighboring sphere was above a certain threshold: below this minimal distance, no sphere was added. The final porosity was controlled by adjusting the minimal distance (i.e., 1.0–1.8 nm) between neighboring spheres and the number of spheres placed into the cylinder. For example, to simulate metal segments with *p* = 42%, the minimal center-to-center distance was set to 1.4 nm, which corresponds to a maximum overlap of 0.6 nm between two spheres. The exact volume of Rh and Ru was then determined by integrating over all mesh cells that have the Rh (or Ru) refractive index.

### Monte Carlo Simulations

Monte Carlo simulations were performed using the freely available simulation software Casino v3.3. The incident electron beam was set at 0.16 nm in diameter at the focal point, with a convergence semiangle of 20 mrad. Line scans were acquired along the heterometallic NR length axis with the beam being focused in the center of the NRs. A total of 100 000 electrons were simulated at each point of the line scan. The transmitted electrons were detected with annular dark field (ADF) detectors with the collection angles experimentally used with our STEM (i.e., 75.4–276 mrad). The electron signal obtained on each metal segments was obtained by averaging the counts along the line scan (at least 100 points per metal).

## Results and Discussion

### Synthesis of Porous AuRh and AuRu NRs

Heterometallic segmented NRs made of Au, Rh, and Ru were synthesized via electrochemical deposition within the tubular pores of AAO membranes, as previously described (Figure 1, more details in the Experimental Methods).^5,9,35^

**Figure 2:**
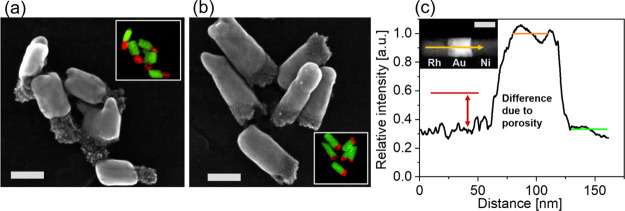
(a,b) Secondary electron STEM images of bimetallic NRs. The inset shows the respective EDX map [Au in green; Rh (Ru) in red]. The scale bars are 50 nm. (a) AuRh. (b) AuRu. (c) Line scan of the intensity of an ADF-STEM image along the length of a RhAuNi NR. The black line shows the relative electron signal intensity measured along the yellow arrow in the inset. The red (Rh), orange (Au), and green (Ni) lines show the intensities obtained from Monte Carlo simulations for the respective metal segments. The scale bar in the inset is 50 nm.

Although the Au and Ni segments are dense, the morphology of the Ru and Rh NRs is porous and nanocrystalline, consisting of nanoparticles with a diameter of ca. 2 nm, as seen in the STEM images (Figure 2a,b). Mallouk et al.^47^ reported that Rh and other high melting point metals preferentially grow via a 3D nucleation–coalescence mechanism, leading to such a nanocrystalline morphology, which is in agreement with our experimental observations.

To characterize the porosity of the Rh and Ru NRs, the electron signals measured at different metal segments using an ADF-STEM detector were compared (Figures 2c and S2c). At high collection angles, the ADF detector collects electrons that have been mostly elastically scattered, with a negligible contribution from Bragg scattering (i.e., diffraction contrast) that occurs at relatively low angles.^48,49^ As such, the contrast observed in ADF-STEM provides direct information of the sample composition, with the electron signal expected to scale as *t*ρ*Z*^α^, where *Z* is the atomic number, *t* is the thickness, ρ is the density at a specific sample’s location, and α is an exponent comprised between 1 and 2 that depends on the detector’s specification (collection angle, i.e., camera length and type of detector) and other experimental factors.^48,50^

To interpret our experimental results, we performed Monte Carlo simulations to model the interaction of the incident electron beam with NRs of similar composition, dimension, and metal sequence using the simulation software Casino v3.3 (Figure S2a, more details on the simulation conditions in the Experimental Methods).^51^ Such simulations provide a good estimate of the collection yield of our ADF detector under our STEM measurement conditions (i.e., accelerating voltage and ADF collection angles). Figure 2c shows a line scan of the intensity measured on a multisegmented RhAuNi NR obtained from an ADF-STEM image. The expected intensities from our Monte Carlo simulations carried out on the same NR (seen in Figure 2c with horizontal red, orange, and green lines) show a good agreement with the experimental data for the Au and the Ni segments, assuming a bulk density (yellow and green lines, respectively). The bulk density of the electrodeposited gold was verified by comparing the ADF-STEM signal intensity measured on electrodeposited Au NRs and on dense Au nanoparticles synthesized via wet chemistry (citrate-protected, Turkevich method).^52,53^ The NRs (diameter = 37 ± 2 nm) and nanoparticles (diameter = 36 ± 3 nm) showed similar intensities (*I*) of *I* = 35 200 ± 965 counts. and *I* = 34 800 ± 2300 counts (data not shown), respectively, clearly indicating that the Au NRs are nonporous and have bulk gold density. However, the intensity of the Rh segment is much lower than that predicted for a dense bulk Rh NR (red line). Because the number of scattered electrons collected using the ADF detector depends on the atomic number and the density of the NRs,^48,50,54^ the Rh porosity was estimated by comparing the intensity obtained from the Monte Carlo simulations and the ADF-STEM images. Yu et al.^54^ used a similar approach to characterize porosity in Pt nanoparticles by comparing the ADF-STEM intensity of dense and porous Pt NPs. The porosities extracted from such measurements were in close agreement with the values determined using ADF-STEM tomography, which validates our approach.^54^ To minimize the influence of variation in NR diameter, the ADF-STEM signal intensity was integrated across the whole NR and averaged over at least 20 different NRs. The background signal arising from the supporting carbon film of the TEM grid was averaged similarly and subtracted from the signal measured on the metals. Since our Monte Carlo simulations showed a linear dependence of the porosity on the electron signal collected using the ADF detector (Figure S2b), a porosity of *p*_Rh_ = 42 ± 10% was estimated for the Rh NRs, where *p*_Rh_ was calculated as
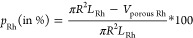
where *V*_porous Rh_ is the volume of Rh present in the porous segment, *R* is the radius of the NR, and π*R*^2^*L*_Rh_ is the volume of the corresponding dense Rh segment (i.e., no porosity). Thus, *p* = 0 corresponds to the bulk density and *p* = 100% corresponds to an empty segment. A similar porosity was obtained for the Ru NRs (*p*_Ru_ = 41 ± 8%, Figure S2c).

For short Rh segments grown at the end of the Au NRs, we observed that our electrodeposition conditions could sometimes lead to Rh segments with a diameter that was slightly smaller than the diameter of the Au segments (Figure 2a). Since the estimation of the metal porosity depends on the diameter,^54^ the Rh porosity was estimated by measuring the ADF-STEM signal on samples where the Rh NRs had a diameter that was similar to the Au NR diameter. This was checked by STEM secondary electron imaging.

### Optical Properties of Porous Rh and Ru NRs

Our preliminary FDTD simulations (not shown) suggest that at *p* = 0, Rh and Ru NRs can sustain longitudinal resonances that can be adjusted by changing the NR aspect ratio.

**Figure 3:**
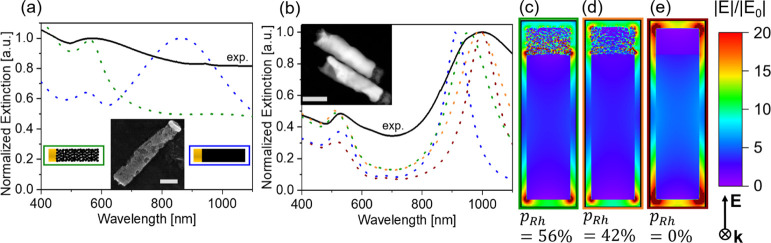
(a) Normalized extinction spectra of a AuRh NR solution (full line). *L*_Au_ = 23 ± 5 nm; *L*_Rh_ = 138 ± 35 nm; and diameter = 43 ± 6 nm and the corresponding simulated spectra (dotted lines) of NRs with similar dimensions. Green: *p*_Rh_ = 42%. Blue: *p*_Rh_ = 0%. The scale bar in the inset corresponds to 50 nm. (b) Normalized extinction spectra of a AuRh NR solution (full black line). *L*_Au_ = 132 ± 10 nm; *L*_Rh_ = 24 ± 6 nm; and diameter: 39 ± 5 nm. Dotted lines: Simulated extinction spectra of AuRh NRs with similar dimensions and various Rh porosities. Blue: *p*_Rh_ = 100% (only Au); green: *p*_Rh_ = 56%; orange: *p*_Rh_ = 42%; and red: *p*_Rh_ = 0% (dense Rh). (c–e) Simulated E-field enhancement maps of the AuRh NRs at 981 nm excitation wavelength. The color of the frame corresponds to the porosity of the Rh segment from (b): (c) green: *p*_Rh_ = 56%, (d) orange: *p*_Rh_ = 42%, and (e) red: *p*_Rh_ = 0%. The direction of propagation and polarization of the incident wave is indicated by the arrow and the cross, respectively. The incident wave vector k propagates into the image plane, and the E-field is polarized along the NR longitudinal axis.

**Figure 4:**
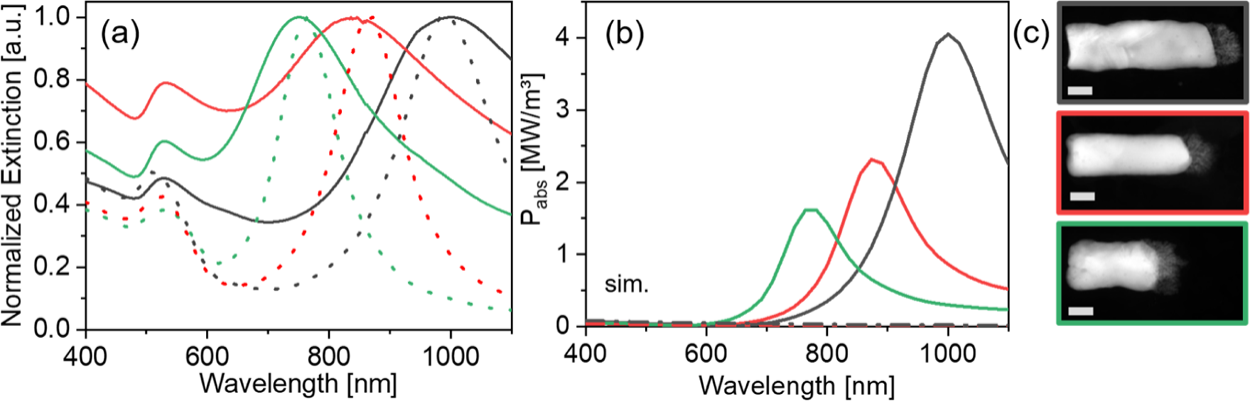
(a) Experimental normalized extinction spectra of AuRu and AuRh NRs (full lines) and the corresponding simulated spectra (dotted lines, *p*_Ru_ = *p*_Rh_ = 42%). Green: AuRu NRs, *L*_Au_ = 93 ± 10 nm; *L*_Ru_ = 18 ± 6 nm; and diameter: 46 ± 6 nm. Red: AuRu NRs; *L*_Au_ = 120 ± 9 nm; *L*_Ru_ = 19 ± 5 nm; and diameter: 46 ± 8 nm. Black: AuRh NRs; *L*_Au_ = 132 ± 10 nm; *L*_Rh_ = 24 ± 6 nm; and diameter: 39 ± 5 nm. (b) Simulated absorbed power density within the Rh (Ru) segment (longitudinal polarization of the incident E-field) of the structures shown in (a). The simulated absorbed power density within the three corresponding isolated Rh and Ru segments is shown with a dashed line (same color code as the bimetallic NRs). (c) ADF-STEM images of the AuRh and AuRu NRs shown in (a). Scale bars are 20 nm.

**Figure 5:**
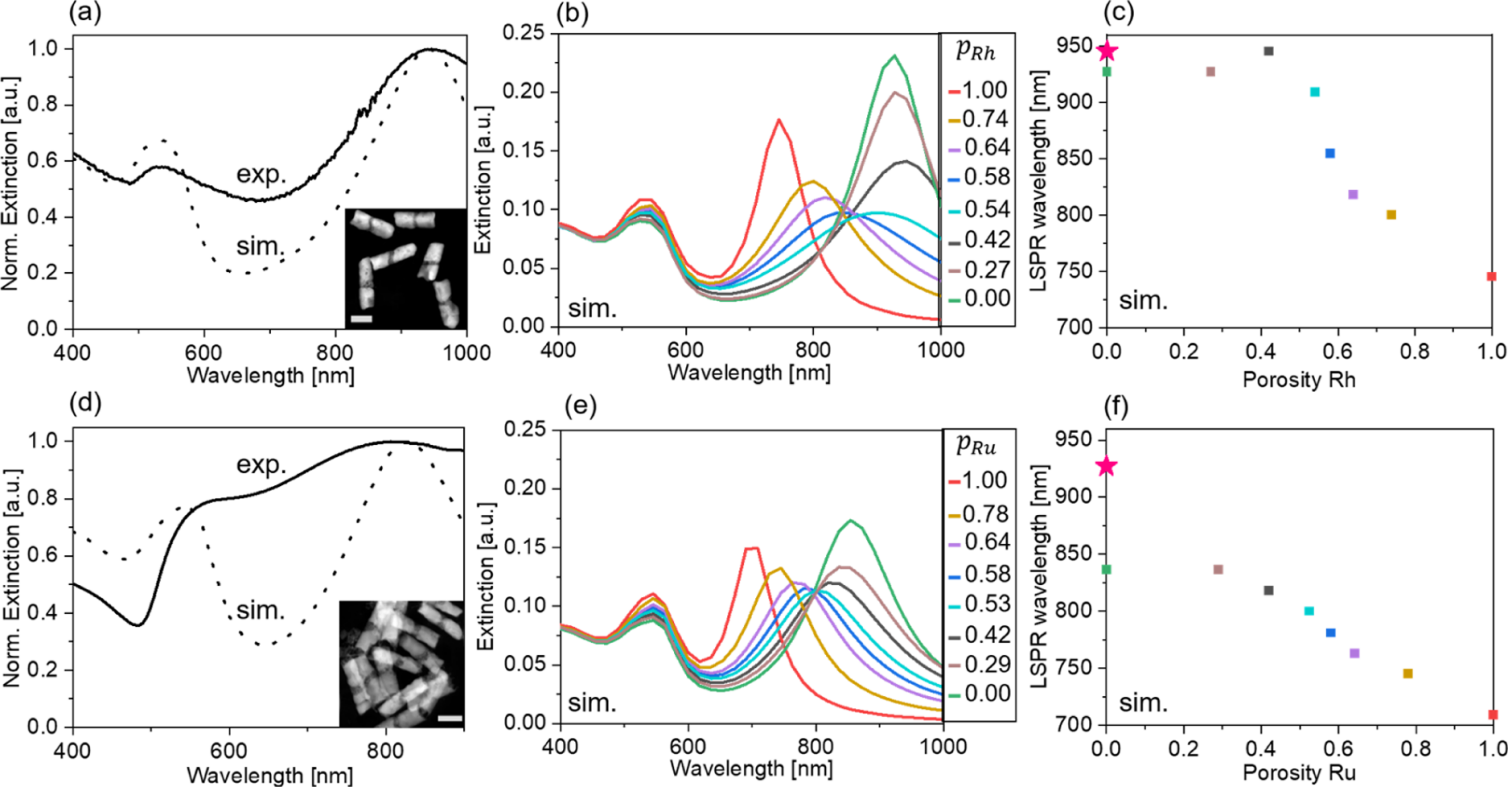
(a) Normalized extinction spectra of a AuRhAu NR solution (full line) with *L*_Au_ = 65 ± 12 nm; *L*_Rh_ = 12 ± 2 nm; and diameter: 41 ± 8 nm and the corresponding simulation (dotted line) of an NR with similar dimensions and *p*_Rh_ = 42%. Scale bar on the inset: 50 nm. (b) Simulated extinction spectra of AuRhAu NRs with different Rh porosities. The same dimensions as the NRs shown in (a). (c) Longitudinal LSPR wavelength of the simulated spectra shown in (b) plotted as a function of *p*_Rh_. As reference, the LSPR wavelength of a Au NR with the same diameter and total length is shown (pink star). (d) Normalized extinction spectra of a AuRuAu NR solution (full line) with *L*_Au_ = 61 ± 8 nm; *L*_Ru_ = 13 ± 4 nm; and diameter: 43 ± 6 nm and the corresponding simulation (dotted line) of a NR with similar dimensions and *p*_Ru_ = 42%. Scale bar on the inset: 50 nm. (e) Simulated extinction spectra of AuRuAu NRs with different Ru porosities. The same dimensions as the NRs shown in (d). (f) Longitudinal LSPR wavelength of the simulated spectra shown in (e) plotted as a function of *p*_Ru_. As reference, the LSPR wavelength of a Au NR with the same total length and diameter is shown (pink star).

Ru shows LSPRs of comparably lower quality. The pure Rh and Ru NRs synthesized in this work did not, however, show any plasmonic behavior most likely because of their porous nature. To investigate how such porous metal segments couple with the well-defined LSPRs of gold NRs, we synthesized AuRh and AuRu (Figures 3 and 4) and AuRhAu and AuRuAu NRs (Figure 5) of varying Au, Rh, and Ru segment lengths, referred to as *L*_Au_, *L*_Rh _, and *L*_Ru_, respectively.

### AuRh NRs with Long Rh Segments (*L*_Rh_ = 6*L*_Au_ ∼ 138 nm)

According to our FDTD simulations, the extinction spectra of such AuRh NRs with *p*_Rh_ = 0 show two well-defined peaks that correspond to the transversal and longitudinal modes (Figure 3a, dashed blue line). The experimental spectrum (solid black line) of the corresponding AuRh NR solution only shows a broad peak at 566 nm, in the 500–800 nm range, suggesting that the Rh segment does not significantly participate in the optical response of the AuRh NR. To match the experimental data, the Rh segment was simulated as a collection of overlapping small nanoparticles (2 nm in diameter, details in the Experimental Methods). At a total Rh porosity of 42%, the position of the simulated spectrum matches fairly well the experimental spectrum, with only one peak around 564 nm (Figure 3a, dashed green line). In comparison, the simulated LSPR of the dense Rh structure peaks at around 854 nm, clearly suggesting that the Rh is not dense. However, the experimental LSPR is broader than the simulated one for *p* = 42% with some contributions in the 600–800 nm range. We attribute this to the deviation of the different metal segment lengths and diameters and of the Rh porosity (Figure 3a), which should yield a collection of LSPRs at different wavelengths, which, when averaged, broaden the resulting spectrum. In particular, based on our STEM measurements, we expect that the Au segment aspect ratio ranges from 0.35 to 1, which should lead to a range of the LSPR peak from 564 to 600 nm. Additionally, deviations in the Rh porosity in the 32–42% range, as expected from our ADF/Monte Carlo analysis, should lead to a more pronounced contribution from the Rh segment in the 600–800 nm range. This suggests that the highly porous nature of the Rh and Ru along with the small size of the nanocrystalline grains (i.e., 2 nm) significantly dampens plasmonic oscillations within the porous lossy segment: due to their porosity and nanocrystalline nature, the long Rh segments cannot sustain an LSPR on their own. Because our FDTD simulations of dense Ru NRs showed low-quality longitudinal LSPRs, porous AuRu NRs of similar dimensions were expected to have ill-defined resonances and were thus not synthesized and studied.

### AuRh NRs with Long Au Segments (*L*_Au_ = 5.6*L*_Rh_ ∼ 130 nm)

The situation is different when a thin porous Rh segment is located at the end of a Au NR. According to our FDTD simulations, the addition of the Rh segment red shifts the longitudinal mode of the reference Au NR (i.e., without Rh, dashed blue curve, Figure 3b), demonstrating that thin porous Rh segments can couple with the Au LSPR. The largest red shift is observed for dense Rh (Figure 3b, dashed red curve), while increasing Rh porosities lead to smaller shifts, which shows that porous Rh segments participate less efficiently in Au plasmonic oscillations (compare the dashed green (*p*_Rh_ = 56%) and orange curves (*p*_Rh_ = 42%) of Figure 3b). Using a porosity of 42%, the simulated extinction spectrum is in good agreement with our experimental results, which again validates our simulation method.

By locating Rh at the end of the plasmonic Au NR, it is also possible to significantly enhance the E-field at the Rh surface. This is clearly seen in Figure 3c–e showing the E-field enhancement maps at 981 nm of AuRh NRs with different porosities. For the highest porosity investigated here (i.e., *p*_Rh_ = 56%, Figure 3c), the E-field hot spots are located at the AuRh interface. At lower porosities (*p*_Rh_ = 42%), the hot spots are also present at the end of the Rh segment (Figure 3d), showing that denser Rh segments couple more efficiently to the Au LSPR, which agrees well with the more pronounced red shift of the longitudinal LSPR at these porosity values. At full density (i.e., *p*_Rh_ = 0%, Figure 3e), the E-field is only enhanced at the end of the AuRh NR. Similar results were obtained on AuRu NRs with long Au segments (*L*_Au_ = 5.2*L*_Ru_ ∼ 93 nm, Figure S3).

By synthesizing AuRh and AuRu NRs with different Au segment lengths, the longitudinal LSPR, and thus the wavelength at which the surface field is most enhanced,^4^ can be precisely adjusted (Figure 4a). Additionally, the E-field inside the porous Rh and Ru segments is also increased around the LSPR wavelength, thus increasing the amount of light absorbed in the porous metal, estimated by calculating the absorbed power density *P*_abs_ (W/m^3^), as shown in Figure 4b.

Our investigation of AuRh and AuRu NRs shows that:

- High porosities, that is, *p*_Rh,Ru_ > 50%, lead to a small red shift and a weak coupling with the Au LSPR, causing an enhanced field that is highly located at the AuRh (Ru) interface.
- Intermediate porosities, 50% > *p*_Rh,Ru_ > 40%, lead to a larger red shift and stronger coupling with the Au LSPR, leading to a field enhancement at the AuRh (Ru) interface and at the end of the Rh (Ru) segment.
- Dense Rh (Ru) segments provide the largest red shift and strongest coupling with the Au LSPR, with a field enhancement that is highly localized at the end of the Rh (Ru) segment.
- It is possible to enhance the bulk absorption within the Rh and Ru metal segment at the LSPR

### AuRhAu NRs (*L*_Au_ = 5.3*L*_Rh_ ∼ 65 nm)

To study further how porous Rh and Ru can couple with a Au plasmonic NR, we prepared and studied Au NR dimers bridged with a thin porous Rh segment (*L*_Rh_ ∼ 12 nm, Figure 5a–c). It was previously reported that dense lossy metals, such as Ni, can electrically bridge two gold NRs, where the AuNiAu NR shows an overall extinction behavior that is similar to a full Au NR with the same dimensions.^55^ The effect of a porous metal segment has, to our knowledge, never been reported. Figure 5a shows that despite its high porosity and nanocrystalline nature, the synthesized porous Rh segment can effectively couple with the longitudinal plasmonic oscillations of the Au NRs (Figure 5a, solid line). This is in good agreement with our FDTD simulations that show two well-defined modes for such AuRhAu NRs with *p*_Rh_ = 42% (Figure 5a, dashed line). As *p*_Rh_ decreases, the longitudinal LSPR wavelength of the AuRhAu NR, λ_AuRhAu_, approaches the expected resonance wavelength of the pure dense Au NR (same diameter and total length), λ_Au_: At *p*_Rh_ = 0, λ_AuRhAu_ = 927 nm, while λ_Au_ = 946 nm (Figure 5c). The simulated spectra of structures with different Rh porosities (Figure 5b) show that a certain density is necessary to efficiently couple with the Au NRs. At *p*_Rh_ > 50%, the Rh porosity does not provide enough conductivity and the resulting AuRhAu NR begins to behave as two Au NRs separated with a dielectric gap (i.e., an Au NR dimer), with a significant blue shift of the longitudinal mode.^35^ This is also evidenced by the fact that the longitudinal LSPR intensity is largely influenced by the Rh porosity (Figures 5b and S4): the introduction of a porous segment between the two isolated Au NRs (i.e., *p*_Rh_ = 100%) leads to a decrease in LSPR intensity with decreasing porosity. At *p*_Rh_ ∼ 50%, the LSPR intensity reaches its minimum. At *p*_Rh_ ≲ 50%, the LSPR intensity increases again, reaching 81% of the LSPR intensity of Au NRs with the same dimensions at *p*_Rh_ = 0%. Thus, a sufficient amount of Rh is necessary to efficiently couple the Au NRs, suggesting that the porous Rh segment acts similarly to a narrow Au segment bridging two Au nanowires: When the Au segment is too thin, the connected structure behaves as a gapped Au nanowire dimer instead of a continuous gold wire.^56^ Additionally, at lower porosity (i.e., 42%), a small additional red shift of the longitudinal mode is observed compared to AuRhAu NRs composed of dense Rh (*p*_Rh_ = 0%, Figure 5c), which is reminiscent of previous reports on porous gold nanostructures that showed similar red shifts.^44,57,58^ Hence, our simulation results show that the porosity of thin Rh segments with nanocrystalline grains can be used to finely tune both the LSPR wavelength position—either in the blue (high porosity) or in the red direction (low porosity)—and intensity.

### AuRuAu NRs (*L*_Au_ = 4.7*L*_Ru_ ∼ 61 nm)

Similar results were obtained with AuRuAu NRs (Figure 5d–f). With *p*_Ru_ = 42%, the simulated spectra predict the spectral position of the extinction peaks of the AuRuAu NR solution quite well (Figure 5d). However, compared to the AuRhAu NRs, the longitudinal mode is less intense, leading to a more ill-defined extinction spectrum. This is because of the Ru dielectric constant above ca. 600 nm, which has a positive real part ε′ and a large ε″ (see Figure S1a). As a result, Ru couples less effectively to the Au dimer than Rh, and λ_AuRuAu_ is significantly blue-shifted compared to λ_Au_ of a dense Au NR with identical dimensions: Even at *p*_Ru_ = 0, λ_AuRuAu_ = 836 nm, while λ_Au_ = 927 nm (Figure 5f). Based on our simulation results, the influence of porosity on the longitudinal LSPR intensity is expected to be similar for both AuRhAu and AuRhAu NRs (Figures 5b,e and S4).

### Spatioselective Enhancement of the Surface and Bulk E-Field

**Figure 6:**
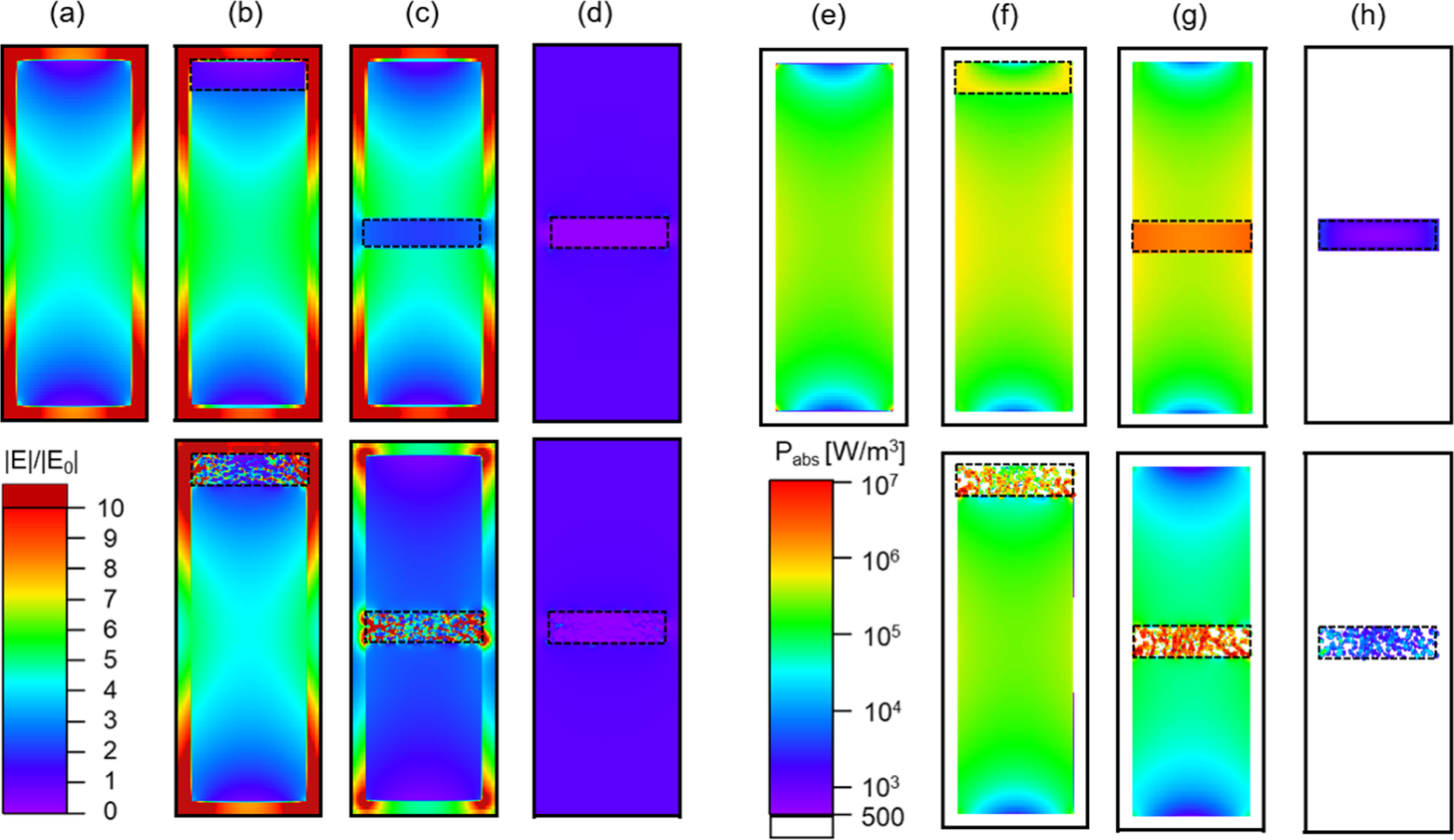
Simulated E-field enhancement maps (a–d) and simulated absorbed power density maps (e–h) of heterometallic NRs at longitudinal polarization at 818 nm excitation wavelength. In the upper row, the maps of a pure Au NR (a,e), a AuRh NR (b,f), a AuRhAu NR (c,g), and a Rh nanodisk (d,h) are shown; the Rh is dense (i.e., *p* = 0%). In the lower row, the same NR structures are shown except that the Rh segment is 42% porous. All NRs have a diameter of 40 nm, and the length of the Rh segments is set to 10 nm. The total length of the NRs shown in (a–c) and (e–g) is 120 nm. The location of the Rh segment is outlined by the dotted black lines.

Because Rh and Ru have been used as photocatalysts,^37,38^ we investigated the influence of the Rh and Ru segment position on the bulk absorption and surface E-field (Figures 6 and S5). Interestingly, *P*_abs_ (i.e., bulk E-field) is expected to be the largest when Rh and Ru are located in the middle of the Au NR dimer (Figures 6g and S5g). This is because the bulk E-field enhancement at the longitudinal LSPR wavelength reaches its maximum in the center of the Au rod (Figures 6a and S5a). This is different from the surface E-field that is the highest at the edges of the Au NR (Figure 6a). Thus, our electromagnetic simulations predict that locating the lossy absorbing segment in the center of the Au NR dimer maximizes bulk absorption (Figures 6g and S5g, respectively). These expected enhancements are much lower when the Rh and Ru segments are located at the end of the NR instead, compared to Figures 6b,f and S4b,f, respectively. Interestingly, our results show that a Rh (Ru) segment with *p* = 42% behaves similarly (Figure 6 lower row and Figure S5 lower row, respectively). Locating Rh (Ru) in the middle of the Au rod is expected to lead to the highest bulk absorption. However, thanks to the porosity, plasmonic hot spots form in the gaps between the Rh (Ru) spheres, which also lead to a significant enhancement of the surface E-field. That is not the case with the dense Rh (Ru) structures.

**Figure 7:**
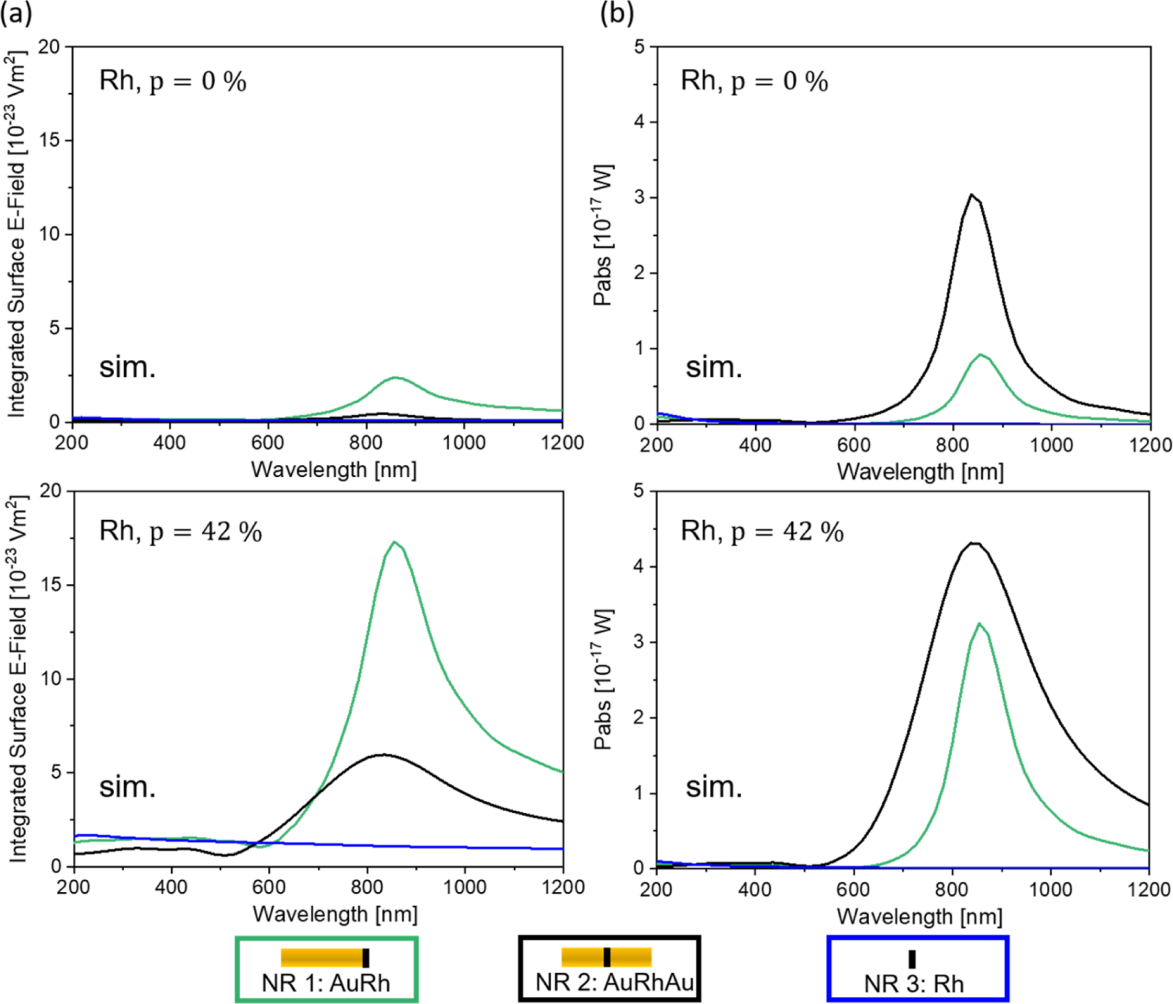
Integrated surface E-field (a) and absorbed power (b) around and within the Rh segments as a function of wavelength. Two different positions within the bimetallic NR were investigated: the end of the Au NR (green, NR 1) and the middle of the Au NR (black, NR 2); for reference, the data for individual segments without Au are shown in blue (NR 3). Two different porosities were investigated: a dense segment with *p* = 0% (upper row) and a porous segment with *p* = 42% (lower row).

To gain a better understanding of the influence of the catalyst position and porosity on the surface E-field and bulk absorption, we integrated the absorbed power density in the Rh (Ru) over the catalyst volume and the E-field over the volume located within a 1 nm region above the Rh (Ru) surface. This provides a direct way to evaluate the surface E-field and *P*_abs_ depending on the catalyst position, porosity, and incident photon wavelength. The data are shown in Figures 7 and S6 for Rh and Ru, respectively, where the upper row corresponds to the dense segment and the lower row corresponds to the porous segment. For a dense Rh (Ru) segment, it is possible to independently enhance the surface E-field or the bulk absorption by locating the Rh (Ru) segment either at the end of the NR or in the center of the Au NR (see Tables 1 and S1 for the expected enhancement values of the surface E-field and the absorbed power for each NR composition and geometry). Indeed, locating the dense Rh (Ru) segment at the end of the Au rod leads to the highest surface E-field enhancement, expected to be 5.6 (11) times larger than when the segment is in the middle of the Au NR. Locating the Rh (Ru) segment in the middle of the Au rod yields the highest *P*_abs_ enhancement, expected to be 3.2 (2.4) times larger than when the Rh (Ru) segment is at the end of the NR.

**Table 1:** Maximum Surface E-Field and Absorbed Power Enhancement within Rh Segments as a Function of Its Porosity and Location within the Bimetallic NRs

	maximum surface E-field enhancement versus individual dense Rh disc			maximum absorbed power enhancement versus individual dense Rh disc		
porosity	Rh	AuRhAu	AuRh	Rh	AuRhAu	AuRh
*p* = 0%	1	5	28	1	1355	425
*p* = 42%	13	69	202	7	2007	1494

A similar trend is observed for porous Rh (Figure 7, lower row) and Ru segments (Figure S6), although with some important differences. Our simulation results suggest that the porous nature of the Rh (Ru) segment provides E-field and *P*_abs_ enhancements that are significantly larger than those provided by a dense Rh (Ru) segment (see Tables 1 and S1 and Figures 7 and S6). Additionally, even when the porous Rh (Ru) segment is located in the middle of the NR, the surface E-field enhancement is large, which is not the case when a dense Rh (Ru) segment is used. Indeed, the expected surface E-field and *P*_abs_ enhancements are 13.8 and 1.5 times larger, respectively, for AuRhAu with a porous Rh segment (*p* = 42%) compared to NRs with a dense Rh segment (*p* = 0%). The largest surface E-field enhancement is expected for porous AuRh NRs, with an enhancement relative to the isolated dense Rh segment of 202 (equal to a |*E*|^2^ enhancement of 40 804), while the largest *P*_abs_ enhancement is expected to be obtained for porous AuRhAu NRs, with an enhancement relative to the isolated dense Rh segment of 2007. The expected enhancements are thus quite significant, supporting such heterostructures to be great platforms for plasmonic catalysis, expected to depend on both the surface and bulk values of |*E*|^2^.^2,14,23^

Overall, our simulation results suggest the following:

- Light absorption and surface E-field can be controlled independently by locating the catalyst either in the middle or at the end of the Au NR.
- Light absorption in the lossy segment is the highest when it is located in the middle of the Au NR.
- A porous segment significantly increases both the surface E-field and light absorption compared to a dense segment.
- It is possible to obtain a large enhancement of the E-field even when the lossy segment is located in the middle of the Au NR, if it has a high enough porosity. This is not the case for a dense segment that shows low surface field enhancement when it is located in the middle of the Au NR.

Because the position of the maximum surface field and *P*_abs_ can be tuned by the NR aspect ratio, such heterometallic NRs are attractive platforms for fundamental photocatalytic studies. Here, we have provided results using Au as the plasmonic absorber, which is limited to LSPR in the >530 nm range. Our preliminary simulation results show that this could be shifted to resonances below 500 nm by incorporating the Rh and Ru catalysts within silver NRs, which could be synthesized using a similar templated approach.^35^

## Conclusions

Our results demonstrate that the bulk and surface E-field of lossy Rh and Ru segments can be selectively enhanced using Au NRs by controlling their porosity and position within the bimetallic NR. The wavelengths at which the fields are most enhanced can be tuned by adjusting the NR composition and dimensions. Such porous lossy thin metal segments can effectively bridge the longitudinal LSP oscillations of Au NR dimers at a minimum porosity of 40%. Additionally, they yield a larger surface and bulk field enhancement than those obtained with dense segments, which makes them greater candidates for photocatalytic applications.

## Supporting Information Available

Dielectric functions of the investigated metals and additional Monte Carlo and FDTD simulations of heterometallic NRs (PDF)

## References

[ref1] Willets K. A.; Van Duyne R. P. Localized Surface Plasmon Resonance Spectroscopy and Sensing. Annu. Rev. Phys. Chem. 2007, 58, 267–297. 10.1146/annurev.physchem.58.032806.104607. 17067281

[ref2] Brongersma M. L.; Halas N. J.; Nordlander P. Plasmon-induced hot carrier science and technology. Nat. Nanotechnol. 2015, 10, 25–34. 10.1038/nnano.2014.311. 25559968

[ref3] Linic S.; Christopher P.; Ingram D. B. Plasmonic-metal nanostructures for efficient conversion of solar to chemical energy. Nat. Mater. 2011, 10, 911–921. 10.1038/nmat3151. 22109608

[ref4] Kelly K. L.; Coronado E.; Zhao L. L.; Schatz G. C. The Optical Properties of Metal Nanoparticles: The Influence of Size, Shape, and Dielectric Environment. J. Phys. Chem. B 2003, 107, 668–677. 10.1021/jp026731y.

[ref5] Osberg K. D.; Rycenga M.; Bourret G. R.; Brown K. A.; Mirkin C. A. Dispersible surface-enhanced Raman scattering nanosheets. Adv. Mater. 2012, 24, 6065–6070. 10.1002/adma.201202845. 22949389

[ref6] Reyer A.; Prinz A.; Giancristofaro S.; Schneider J.; Bertoldo Menezes D.; Zickler G.; Bourret G. R.; Musso M. E. Investigation of Mass-Produced Substrates for Reproducible Surface-Enhanced Raman Scattering Measurements over Large Areas. ACS Appl. Mater. Interfaces 2017, 9, 25445–25454. 10.1021/acsami.7b06002. 28737921

[ref7] Baffou G.; Quidant R. Thermo-plasmonics: using metallic nanostructures as nano-sources of heat. Laser Photonics Rev. 2013, 7, 171–187. 10.1002/lpor.201200003.

[ref8] Rosi N. L.; Mirkin C. A. Nanostructures in biodiagnostics. Chem. Rev. 2005, 105, 1547–1562. 10.1021/cr030067f. 15826019

[ref9] Bourret G. R.; Ozel T.; Blaber M.; Shade C. M.; Schatz G. C.; Mirkin C. A. Long-range plasmophore rulers. Nano Lett. 2013, 13, 2270–2275. 10.1021/nl400884j. 23594361

[ref10] Ringler M.; Schwemer A.; Wunderlich M.; Nichtl A.; Kürzinger K.; Klar T. A.; Feldmann J. Shaping emission spectra of fluorescent molecules with single plasmonic nanoresonators. Phys. Rev. Lett. 2008, 100, 203002 10.1103/physrevlett.100.203002. 18518528

[ref11] Christopher P.; Xin H.; Linic S. Visible-light-enhanced catalytic oxidation reactions on plasmonic silver nanostructures. Nat. Chem. 2011, 3, 467–472. 10.1038/nchem.1032. 21602862

[ref12] Hou W.; Hung W. H.; Pavaskar P.; Goeppert A.; Aykol M.; Cronin S. B. Photocatalytic Conversion of CO2 to Hydrocarbon Fuels via Plasmon-Enhanced Absorption and Metallic Interband Transitions. ACS Catal. 2011, 1, 929–936. 10.1021/cs2001434.

[ref13] Mukherjee S.; Libisch F.; Large N.; Neumann O.; Brown L. V.; Cheng J.; Lassiter J. B.; Carter E. A.; Nordlander P.; Halas N. J. Hot electrons do the impossible: plasmon-induced dissociation of H2 on Au. Nano Lett. 2013, 13, 240–247. 10.1021/nl303940z. 23194158

[ref14] Besteiro L. V.; Govorov A. O. Amplified Generation of Hot Electrons and Quantum Surface Effects in Nanoparticle Dimers with Plasmonic Hot Spots. J. Phys. Chem. C 2016, 120, 19329–19339. 10.1021/acs.jpcc.6b05968.

[ref15] Ostovar B.; Cai Y.-Y.; Tauzin L. J.; Lee S. A.; Ahmadivand A.; Zhang R.; Nordlander P.; Link S. Increased Intraband Transitions in Smaller Gold Nanorods Enhance Light Emission. ACS Nano 2020, 14, 15757–15765. 10.1021/acsnano.0c06771. 32852941

[ref16] Manjavacas A.; Liu J. G.; Kulkarni V.; Nordlander P. Plasmon-induced hot carriers in metallic nanoparticles. ACS Nano 2014, 8, 7630–7638. 10.1021/nn502445f. 24960573

[ref17] Zhang Y.; He S.; Guo W.; Hu Y.; Huang J.; Mulcahy J. R.; Wei W. D. Surface-Plasmon-Driven Hot Electron Photochemistry. Chem. Rev. 2018, 118, 2927–2954. 10.1021/acs.chemrev.7b00430. 29190069

[ref18] Aslam U.; Chavez S.; Linic S. Controlling energy flow in multimetallic nanostructures for plasmonic catalysis. Nat. Nanotechnol. 2017, 12, 1000–1005. 10.1038/nnano.2017.131. 28737751

[ref19] Aslam U.; Rao V. G.; Chavez S.; Linic S. Catalytic conversion of solar to chemical energy on plasmonic metal nanostructures. Nat. Catal. 2018, 1, 656–665. 10.1038/s41929-018-0138-x.

[ref20] Olsen T.; Schiøtz J. Origin of power laws for reactions at metal surfaces mediated by hot electrons. Phys. Rev. Lett. 2009, 103, 238301 10.1103/physrevlett.103.238301. 20366180

[ref21] Christopher P.; Xin H.; Marimuthu A.; Linic S. Singular characteristics and unique chemical bond activation mechanisms of photocatalytic reactions on plasmonic nanostructures. Nat. Mater. 2012, 11, 1044–1050. 10.1038/nmat3454. 23178296

[ref22] Wang F.; Li C.; Chen H.; Jiang R.; Sun L.-D.; Li Q.; Wang J.; Yu J. C.; Yan C.-H. Plasmonic harvesting of light energy for Suzuki coupling reactions. J. Am. Chem. Soc. 2013, 135, 5588–5601. 10.1021/ja310501y. 23521598

[ref23] Hartland G. V.; Besteiro L. V.; Johns P.; Govorov A. O. What’s so Hot about Electrons in Metal Nanoparticles?. ACS Energy Lett. 2017, 2, 1641–1653. 10.1021/acsenergylett.7b00333.

[ref24] Dubi Y.; Un I. W.; Sivan Y. Thermal effects - an alternative mechanism for plasmon-assisted photocatalysis. Chem. Sci. 2020, 11, 5017–5027. 10.1039/c9sc06480j. 34122958 PMC8159236

[ref25] Zhan C.; Wang Q. X.; Yi J.; Chen L.; Wu D. Y.; Wang Y.; Xie Z. X.; Moskovits M.; Tian Z. Q. Plasmonic nanoreactors regulating selective oxidation by energetic electrons and nanoconfined thermal fields. Sci. Adv. 2021, 7, eabf0962 10.1126/sciadv.abf0962. 33674315 PMC7935359

[ref26] Jain P. K. Taking the Heat Off of Plasmonic Chemistry. J. Phys. Chem. C 2019, 123, 24347–24351. 10.1021/acs.jpcc.9b08143.

[ref27] Baffou G.; Bordacchini I.; Baldi A.; Quidant R. Simple experimental procedures to distinguish photothermal from hot-carrier processes in plasmonics. Light: Sci. Appl. 2020, 9, 108 10.1038/s41377-020-00345-0. 32612818 PMC7321931

[ref28] Zhang X.; Li X.; Reish M. E.; Zhang D.; Su N. Q.; Gutiérrez Y.; Moreno F.; Yang W.; Everitt H. O.; Liu J. Plasmon-Enhanced Catalysis: Distinguishing Thermal and Nonthermal Effects. Nano Lett. 2018, 18, 1714–1723. 10.1021/acs.nanolett.7b04776. 29438619

[ref29] Penner R. M.; Martin C. R. Preparation and electrochemical characterization of ultramicroelectrode ensembles. Anal. Chem. 2002, 59, 2625–2630. 10.1021/ac00148a020.

[ref30] Martin C. R. Nanomaterials: a membrane-based synthetic approach. Science 1994, 266, 1961–1966. 10.1126/science.266.5193.1961. 17836514

[ref31] Routkevitch D.; Bigioni T.; Moskovits M.; Xu J. M. Electrochemical Fabrication of CdS Nanowire Arrays in Porous Anodic Aluminum Oxide Templates. J. Phys. Chem. 1996, 100, 14037–14047. 10.1021/jp952910m.

[ref32] Qin L.; Park S.; Huang L.; Mirkin C. A. On-wire lithography. Science 2005, 309, 113–115. 10.1126/science.1112666. 15994551

[ref33] Ozel T.; Bourret G. R.; Mirkin C. A. Coaxial lithography. Nat. Nanotechnol. 2015, 10, 319–324. 10.1038/nnano.2015.33. 25799520

[ref34] Wendisch F. J.; Saller M. S.; Eadie A.; Reyer A.; Musso M.; Rey M.; Vogel N.; Diwald O.; Bourret G. R. Three-Dimensional Electrochemical Axial Lithography on Si Micro- and Nanowire Arrays. Nano Lett. 2018, 18, 7343–7349. 10.1021/acs.nanolett.8b03608. 30359028 PMC6238956

[ref35] Osberg K. D.; Schmucker A. L.; Senesi A. J.; Mirkin C. A. One-dimensional nanorod arrays: independent control of composition, length, and interparticle spacing with nanometer precision. Nano Lett. 2011, 11, 820–824. 10.1021/nl1041534. 21226511

[ref36] Ozel T.; Ashley M. J.; Bourret G. R.; Ross M. B.; Schatz G. C.; Mirkin C. A. Solution-Dispersible Metal Nanorings with Deliberately Controllable Compositions and Architectural Parameters for Tunable Plasmonic Response. Nano Lett. 2015, 15, 5273–5278. 10.1021/acs.nanolett.5b01594. 26133945

[ref37] Zhang X.; Li X.; Zhang D.; Su N. Q.; Yang W.; Everitt H. O.; Liu J. Product selectivity in plasmonic photocatalysis for carbon dioxide hydrogenation. Nat. Commun. 2017, 8, 14542 10.1038/ncomms14542. 28230100 PMC5348736

[ref38] Kim C.; Hyeon S.; Lee J.; Kim W. D.; Lee D. C.; Kim J.; Lee H. Energy-efficient CO2 hydrogenation with fast response using photoexcitation of CO2 adsorbed on metal catalysts. Nat. Commun. 2018, 9, 3027 10.1038/s41467-018-05542-5. 30072704 PMC6072744

[ref39] Palik E. D. Handbook of Optical Constants of Solids; Academic Press: San Diego, 1997; Vol. II.

[ref40] Palik E. Handbook of Optical Constants of Solids; Academic Press: San Diego, 1998; Vol. III.

[ref41] Johnson P. B.; Christy R. W. Optical Constants of the Noble Metals. Phys. Rev. B: Solid State 1972, 6, 4370–4379. 10.1103/physrevb.6.4370.

[ref42] Koya A. N.; Zhu X.; Ohannesian N.; Yanik A. A.; Alabastri A.; Proietti Zaccaria R.; Krahne R.; Shih W.-C.; Garoli D. Nanoporous Metals: From Plasmonic Properties to Applications in Enhanced Spectroscopy and Photocatalysis. ACS Nano 2021, 15, 6038–6060. 10.1021/acsnano.0c10945. 33797880 PMC8155319

[ref43] Zhang Q.; Large N.; Nordlander P.; Wang H. Porous Au Nanoparticles with Tunable Plasmon Resonances and Intense Field Enhancements for Single-Particle SERS. J. Phys. Chem. Lett. 2014, 5, 370–374. 10.1021/jz402795x. 26270713

[ref44] Schubert I.; Huck C.; Kröber P.; Neubrech F.; Pucci A.; Toimil-Molares M. E.; Trautmann C.; Vogt J. Porous Gold Nanowires: Plasmonic Response and Surface-Enhanced Infrared Absorption. Adv. Opt. Mater. 2016, 4, 1838–1845. 10.1002/adom.201600430.

[ref45] Zheng B. Y.; Zhao H.; Manjavacas A.; McClain M.; Nordlander P.; Halas N. J. Distinguishing between plasmon-induced and photoexcited carriers in a device geometry. Nat. Commun. 2015, 6, 7797 10.1038/ncomms8797. 26165521 PMC4510964

[ref46] Swearer D. F.; Zhao H.; Zhou L.; Zhang C.; Robatjazi H.; Martirez J. M. P.; Krauter C. M.; Yazdi S.; McClain M. J.; Ringe E.; Carter E. A.; Nordlander P.; Halas N. J. Heterometallic antenna-reactor complexes for photocatalysis. Proc. Natl. Acad. Sci. U.S.A. 2016, 113, 8916–8920. 10.1073/pnas.1609769113. 27444015 PMC4987788

[ref47] Tian M.; Wang J.; Kurtz J.; Mallouk T. E.; Chan M. H. W. Electrochemical Growth of Single-Crystal Metal Nanowires via a Two-Dimensional Nucleation and Growth Mechanism. Nano Lett. 2003, 3, 919–923. 10.1021/nl034217d. 27676178

[ref48] Treacy M. M. J. Z dependence of electron scattering by single atoms into annular dark-field detectors. Microsc. Microanal. 2011, 17, 847–858. 10.1017/s1431927611012074. 22051035

[ref49] Treacy M. M. J.; Howie A.; Wilson C. J. Z contrast of platinum and palladium catalysts. Philos. Mag. A 1978, 38, 569–585. 10.1080/01418617808239255.

[ref50] Yamashita S.; Kikkawa J.; Yanagisawa K.; Nagai T.; Ishizuka K.; Kimoto K. Atomic number dependence of Z contrast in scanning transmission electron microscopy. Sci. Rep. 2018, 8, 12325 10.1038/s41598-018-30941-5. 30120323 PMC6098135

[ref51] Drouin D.; Couture A. R.; Joly D.; Tastet X.; Aimez V.; Gauvin R. CASINO V2.42: a fast and easy-to-use modeling tool for scanning electron microscopy and microanalysis users. Scanning 2007, 29, 92–101. 10.1002/sca.20000. 17455283

[ref52] Turkevich J.; Stevenson P. C.; Hillier J. A study of the nucleation and growth processes in the synthesis of colloidal gold. Discuss. Faraday Soc. 1951, 11, 55–75. 10.1039/df9511100055.

[ref53] Goulet P. J. G.; Bourret G. R.; Lennox R. B. Facile phase transfer of large, water-soluble metal nanoparticles to nonpolar solvents. Langmuir 2012, 28, 2909–2913. 10.1021/la2038894. 22283327

[ref54] Yu W.; Batchelor-McAuley C.; Wang Y.-C.; Shao S.; Fairclough S. M.; Haigh S. J.; Young N. P.; Compton R. G. Characterising porosity in platinum nanoparticles. Nanoscale 2019, 11, 17791–17799. 10.1039/c9nr06071e. 31552997

[ref55] Kim S.; Shuford K. L.; Bok H.-M.; Kim S. K.; Park S. Intraparticle surface plasmon coupling in quasi-one-dimensional nanostructures. Nano Lett. 2008, 8, 800–804. 10.1021/nl0726353. 18254604

[ref56] Schnell M.; García-Etxarri A.; Huber A. J.; Crozier K.; Aizpurua J.; Hillenbrand R. Controlling the near-field oscillations of loaded plasmonic nanoantennas. Nat. Photonics 2009, 3, 287–291. 10.1038/nphoton.2009.46.

[ref57] Vidal C.; Wang D.; Schaaf P.; Hrelescu C.; Klar T. A. Optical Plasmons of Individual Gold Nanosponges. ACS Photonics 2015, 2, 1436–1442. 10.1021/acsphotonics.5b00281. 26523285 PMC4616225

[ref58] Rao W.; Wang D.; Kups T.; Baradács E.; Parditka B.; Erdélyi Z.; Schaaf P. Nanoporous Gold Nanoparticles and Au/Al2O3 Hybrid Nanoparticles with Large Tunability of Plasmonic Properties. ACS Appl. Mater. Interfaces 2017, 9, 6273–6281. 10.1021/acsami.6b13602. 28145115

