# Supplementary material for: Selective Enhancement of Surface and Bulk E-Field within Porous AuRh and AuRu Nanorods
Source: J Phys Chem C Nanomater Interfaces. 2021 Dec 12;125(50):27661–70. doi: 10.1021/acs.jpcc.1c08699 (PMC8713288; doi:10.1021/acs.jpcc.1c08699)
Supplement: Supplementary file 1 — jp1c08699_si_001.pdf [file jp1c08699_si_001.pdf]

Supporting Information

Selective Enhancement of Surface and Bulk E-Field within Porous AuRh and AuRu Nanorods

*Joshua Piaskowski, Alisher Ibragimov, Fedja J. Wendisch and Gilles R. Bourret**

Department of Chemistry and Physics of Materials, University of Salzburg, Jakob Haringer

Strasse 2A, A-5020 Salzburg, Austria

Email: gilles.bourret@plus.sbg.ac.at

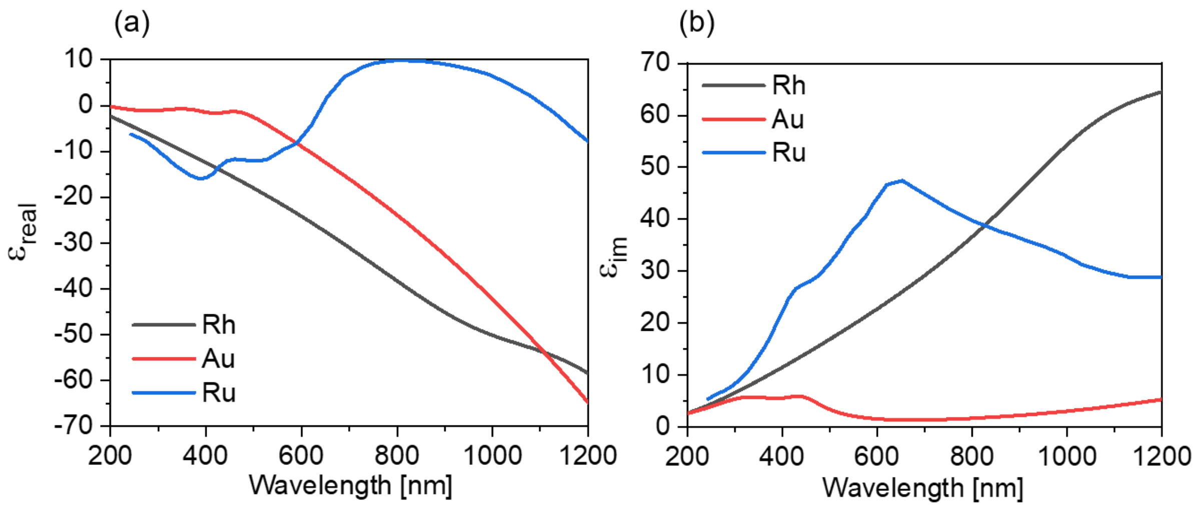

Figure S1: Real (a) and imaginary (b) part of the dielectric function of Rh,[1] Ru,[2] and Au.[3]

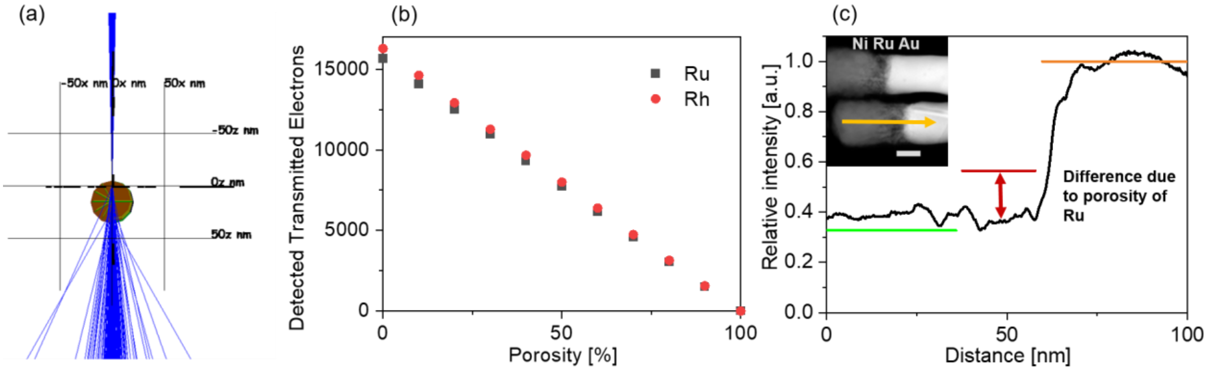

Figure S2: (a) Typical set up of a Monte Carlo simulation. 100000 incident electrons are focused on the center of the NR and scattered by it. Using the annular dark-field sensor the electrons scattered in a certain angular range can be detected. (b) Detected transmitted electrons (100,000 incident electrons; 75.4-276 mrad collection angle) obtained from Monte Carlo simulations of Rh and Ru NRs with 45 nm diameter and different porosities. The porosity was controlled by adjusting the density of the respective metal in the materials set up. (c) Line scan of the intensity of an ADF-STEM image along the length of a NiRuAu NR. The black line shows the relative electron signal intensity measured along the yellow arrow in the inset. The red (Ru), orange (Au), green (Ni) line show the intensities obtained from Monte Carlo simulations for the respective metal segments. The scale bar in the inset corresponds to 20 nm.

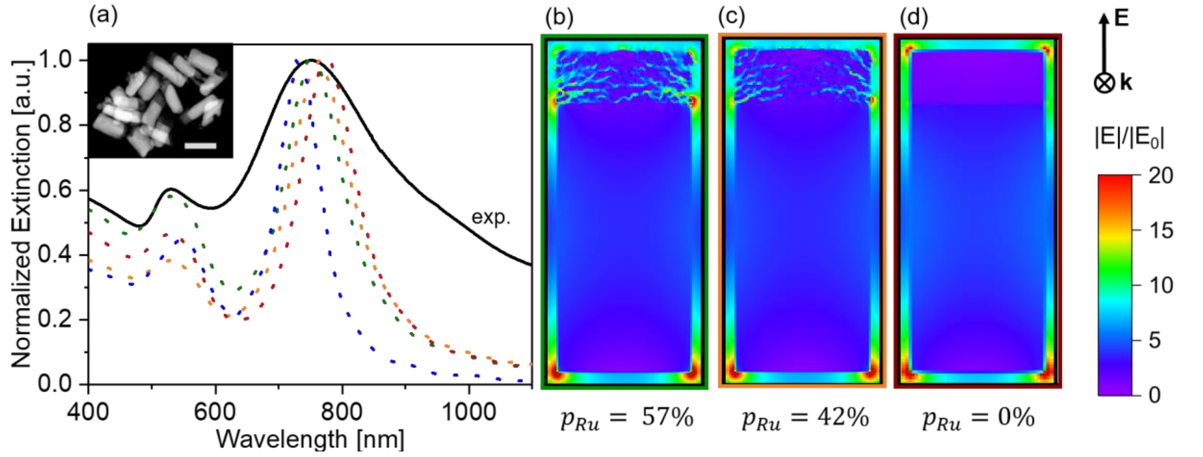

Figure S3: (a) Normalized extinction spectra of a AuRu NR solution (full line). Au length 93 ± 10 nm; Ru length 18 ± 6 nm; diameter 46 ± 6 nm. Simulated extinction spectra of AuRu NRs with dimensions comparable to the NR synthesized, with varying Ru porosity (dotted lines). (Blue: $p_{Ru} = 100$ % (only Au); Green: $p_{Ru} = 57$ %, orange: $p_{Ru} = 42$ %; red: $p_{Ru} = 0$ % (dense Ru)) (b)-(d): E-Field enhancement maps of the AuRu NRs at 764 nm excitation wavelength. The color of the frame corresponds to the porosity of the Rh segment from (a): (b) Green: $p_{Ru} = 57$ %, (c) orange: $p_{Ru} = 42$ % and (d) red: $p_{Ru} = 0$ %. The direction of propagation and polarization of the incident wave is indicated by the arrow and the cross, respectively. The incident wave vector k propagates into the image plane, the E-field is polarized along the NR length axis.

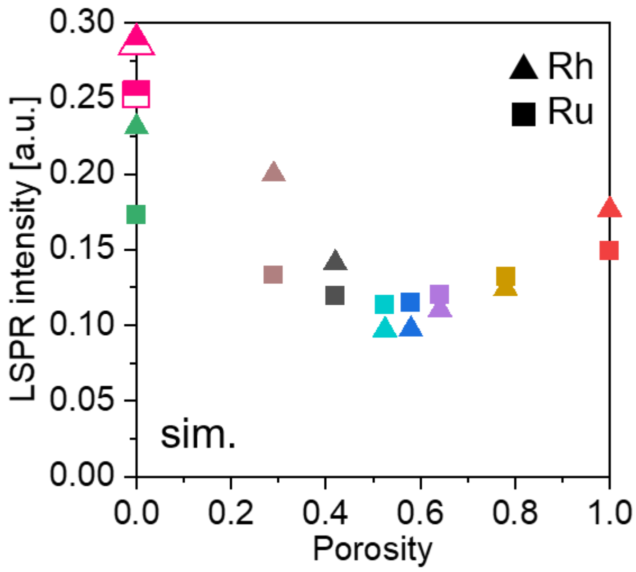

Figure S4: (a) Longitudinal LSPR intensity of NRs shown in Figure 5 (b ,e) as a function of porosity. The AuRhAu NRs are marked with triangles, the AuRuAu NRs are marked with squares. As reference, the LSPR intensity of two Au NRs with the same total length and diameter are shown (a pink half colored triangle corresponding to the dimensions of the AuRhAu NRs and a pink half colored square corresponding to the dimensions of the AuRuAu NRs).

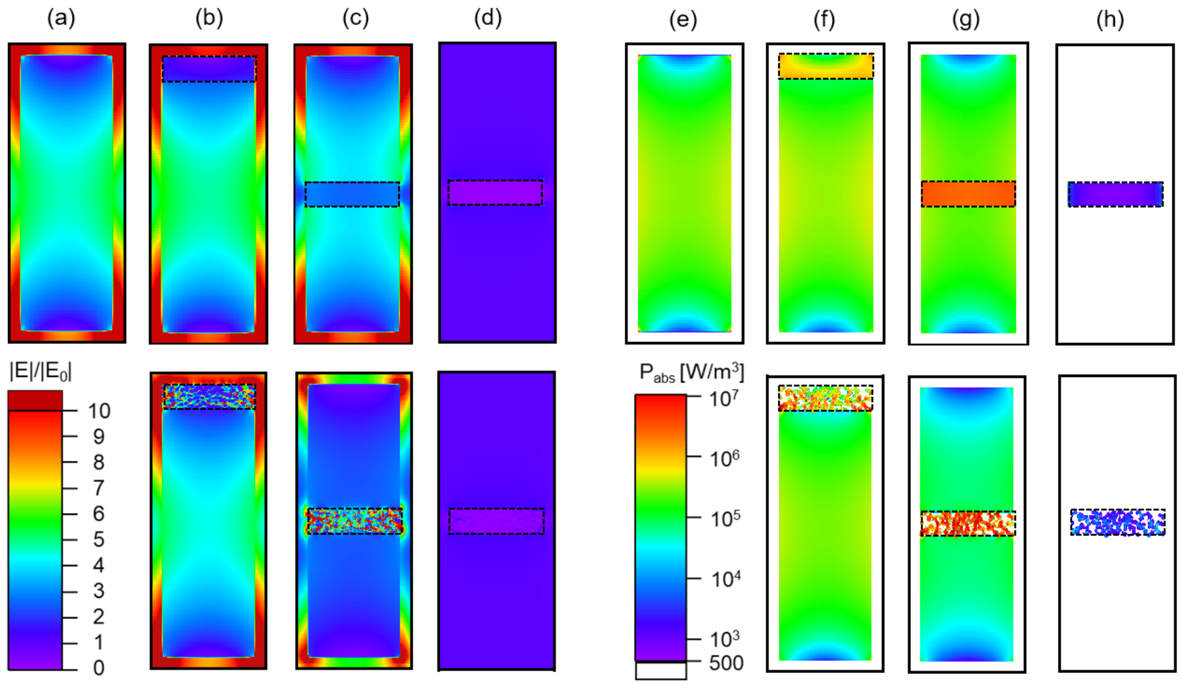

Figure S5: Simulated E-Field enhancement maps (a-d) and simulated absorbed power maps (e-h) of heterometallic NRs at longitudinal polarization at 818 nm excitation wavelength. In the upper row the maps of a pure Au NR (a, e), an AuRu NR (b, f), an AuRuAu NR (c, g) and a Ru NR (d, h) are shown, the Ru is dense. In the lower row the same NRs are shown except that the Ru segment is 42% porous. (All NRs have a diameter of 40 nm, the length of the Ru segments is set to 10 nm. The total length of the NRs shown in (a-c) and (e-g) is 120 nm.

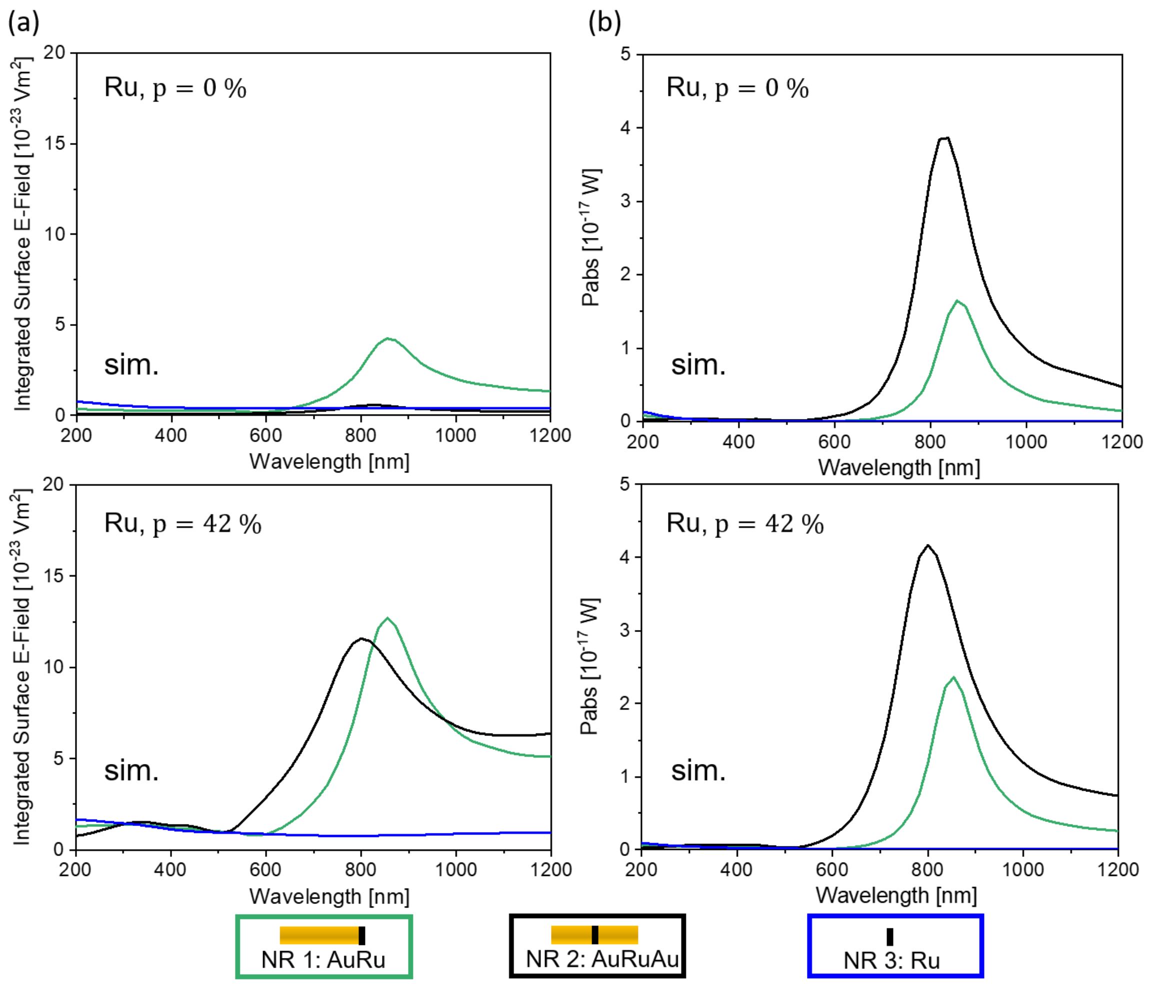

Figure S6: Integrated surface E-field (a) and absorbed power (b) around and within the Ru segments as function of wavelength. Two different positions within the bimetallic NR were investigated: the end of the Au NR (green, NR 1) and the middle of the Au NR (black, NR 2) for reference the data for individual segments without Au are shown in blue (NR 3). Two different porosities were investigated: a dense segment with p = 0 % (upper row) and a porous segment with p = 42 % (lower row).

Table S1: Maximum surface E-field and absorbed power enhancement within Ru segments as a function of its porosity and location within the heterometallic NRs.

	Maximum surface E-field enhancement vs. individual dense Ru disc			Maximum absorbed power enhancement vs. individual dense Ru disc		
Porosity	Ru	AuRuAu	AuRu	Ru	AuRuAu	AuRu
p = 0 %	1	1	11	1	960	400
p = 42 %	11	29	32	5	1057	573

References

1. Palik, E. D. *Handbook of Optical Constants of Solids*. Academic Press: San Diego, **1997**; Vol. II.
2. Palik, E. *Handbook of Optical Constants of Solids*. Academic Press: San Diego, **1998**; Vol. III.
3. Johnson, P. B.; Christy, R. W. Optical Constants of the Noble Metals. *Phys. Rev. B* **1972**, 6 (12), 4370-4379.
